# Distinct macrophage phenotypes skewed by local granulocyte macrophage colony‐stimulating factor (GM‐CSF) and macrophage colony‐stimulating factor (M‐CSF) are associated with tissue destruction and intimal hyperplasia in giant cell arteritis

**DOI:** 10.1002/cti2.1164

**Published:** 2020-08-27

**Authors:** William F Jiemy, Yannick van Sleen, Kornelis SM van der Geest, Hilde A ten Berge, Wayel H Abdulahad, Maria Sandovici, Annemieke MH Boots, Peter Heeringa, Elisabeth Brouwer

**Affiliations:** ^1^ Department of Pathology and Medical Biology University of Groningen University Medical Center Groningen Groningen The Netherlands; ^2^ Faculty of Applied Science UCSI University UCSI Heights Cheras, Kuala Lumpur Malaysia; ^3^ Department of Rheumatology and Clinical Immunology University of Groningen University Medical Center Groningen Groningen The Netherlands

**Keywords:** giant cell arteritis, granulocyte macrophage colony‐stimulating factor, macrophages, macrophage colony‐stimulating factor, vasculitis

## Abstract

**Objective:**

To determine the presence and spatial distribution of different macrophage phenotypes, governed by granulocyte macrophage colony‐stimulating factor (GM‐CSF) and macrophage colony‐stimulating factor (M‐CSF) skewing signals, in giant cell arteritis (GCA) lesions.

**Methods:**

Temporal artery biopsies (TABs, *n* = 11) from treatment‐naive GCA patients, aorta samples from GCA‐related aneurysms (*n* = 10) and atherosclerosis (*n* = 10) were stained by immunohistochemistry targeting selected macrophage phenotypic markers, cytokines, matrix metalloproteinases (MMPs) and growth factors. *In vitro* macrophage differentiation (*n* = 10) followed by flow cytometry, Luminex assay and ELISA were performed to assess whether GM‐CSF and M‐CSF are drivers of macrophage phenotypic heterogeneity.

**Results:**

A distinct spatial distribution pattern of macrophage phenotypes in TABs was identified. CD206^+^/MMP‐9^+^ macrophages were located at the site of tissue destruction, whereas FRβ^+^ macrophages were located in the inner intima of arteries with high degrees of intimal hyperplasia. Notably, this pattern was also observed in macrophage‐rich areas in GCA aortas but not in atherosclerotic aortas. Flow cytometry showed that GM‐CSF treatment highly upregulated CD206 expression, while FRβ was expressed by M‐CSF‐skewed macrophages, only. Furthermore, localised expression of GM‐CSF and M‐CSF was detected, likely contributing to macrophage heterogeneity in the vascular wall.

**Conclusions:**

Our data document a distinct spatial distribution pattern of CD206^+^/MMP‐9^+^ macrophages and FRβ^+^ macrophages in GCA linked to tissue destruction and intimal proliferation, respectively. We suggest that these distinct macrophage phenotypes are skewed by sequential GM‐CSF and M‐CSF signals. Our study adds to a better understanding of the development and functional role of macrophage phenotypes in the pathogenesis of GCA and opens opportunities for the design of macrophage‐targeted therapies.

## Graphical abstract

In this study, we revealed a distinct spatial distribution of macrophage phenotypes in GCA‐affected vessel walls. CD206^+^/MMP‐9^+^ macrophages were located at the site of tissue destruction, whereas FRβ^+^ macrophages were located in the inner intima of arteries with high degrees of intimal hyperplasia. We suggest that these distinct macrophage phenotypes are skewed by sequential GM‐CSF and M‐CSF signals.

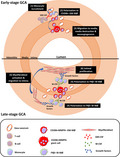

## Introduction

Giant cell arteritis (GCA) is an inflammatory disorder and the most frequent form of vasculitis. GCA affects the medium and large vessels and occurs exclusively in elderly individuals.^1^ Patients with GCA present with various symptoms, depending on which arteries are affected.^2^ Inflammation of cranial arteries (e.g. the temporal artery) often leads to headache but can also cause ischaemic symptoms such as jaw claudication and vision loss. Large arteries such as the aorta can also be affected, although symptoms of large‐vessel GCA are often nonspecific, which may lead to diagnostic delay.^3^ Without proper treatment, large‐vessel GCA can cause aortic aneurysm and dissection as a result of chronic damage to the vascular wall.^4^ Glucocorticoids (GCs) remain the main treatment option for GCA patients, although novel GC‐sparing therapies have recently become available, such as tocilizumab (IL‐6 receptor blockade).^5^

The pathology of GCA is characterised by a granulomatous infiltrate in the vessel wall, which mainly consists of T cells and macrophages.^1^ However, the trigger of this process is still unclear. It is generally thought that resident dendritic cells in the vessel wall, after pattern recognition receptor stimulation, become activated and start producing cytokines and chemokines that attract T cells into the vessel wall. Activated T cells produce cytokines and growth factors that activate the vascular smooth muscle cells, which in turn produce CCL2 and CX3CL1, recruiting monocytes to the lesion. These monocytes differentiate into macrophages upon entering the tissue.^1^, ^6^ Some of the macrophages fuse and develop into multinucleated giant cells.^7^ Macrophages in GCA lesions are derived from circulating monocytes, of which three subsets have been identified: classical CD14^high^CD16^−^ cells, intermediate CD14^high^CD16^+^ cells and non‐classical CD14^dim^CD16^+^ cells.

Macrophages are the main producers of proinflammatory cytokines, growth factors and tissue‐destructive molecules, including matrix metalloproteinases (MMPs),^8^ which enhance inflammation, cause damage to the lamina elastica^9^ and contribute to vessel wall remodelling and intimal hyperplasia. Infiltration and cellular proliferation in the intimal layer of the artery ultimately lead to occlusion, a process responsible for the ischaemic symptoms.^1^ Recently, macrophage‐derived MMP‐9 was reported to be essential for T‐cell infiltration into the vessel wall.^10^ Macrophages also play a major role in the skewing of T cells in the vessel wall by producing polarising cytokines. T cells activated in the presence of IL‐12 and IL‐18 develop into IFNγ‐producing Th1 cells, whereas IL‐1β, IL‐6 and IL‐23 lead to Th17 activation.^11^ GCA tissue displays a mixed population of proinflammatory Th1 and Th17 cells but essentially lacks Th2 cells or Tregs.^1^

Macrophages are incredibly plastic cells that can switch phenotypes and functions depending on environmental cues. The growth factors, granulocyte macrophage colony‐stimulating factor (GM‐CSF) and macrophage colony‐stimulating factor (M‐CSF), were shown to skew macrophages into different phenotypes.^12^ CD206 (mannose receptor), a macrophage marker associated with tissue remodelling, was found to be highly upregulated on GM‐CSF‐primed macrophages.^13^ Alternatively, folate receptor β (FRβ) has been described as a marker of M‐CSF‐induced differentiation.^14^ FRβ^+^ macrophages have been associated with fibroblast activation and proliferation in rheumatoid arthritis.^15^ Fibroblast proliferation is also a key finding in the intimal layer in GCA and eventually leads to luminal occlusion. Whether macrophages from GCA patients respond similarly to GM‐CSF and M‐CSF skewing signals needs to be elucidated. Moreover, the expression of GM‐CSF and M‐CSF in GCA lesions and their relation to macrophage heterogeneity has not yet been assessed.

Although macrophages are one of the dominant inflammatory cellular infiltrates in GCA lesions,^6^, ^16^, ^17^, ^18^, ^19^ little is known about their phenotypic heterogeneity and spatial distribution within the affected vessel wall. We hypothesised that within GCA lesions, distinct macrophage phenotypes are associated with distinct functions and lesion morphology, dictated by local GM‐CSF and M‐CSF production. To address this hypothesis, we first comprehensively characterised macrophage phenotypes in affected temporal artery biopsies (TABs) and aortic samples from GCA patients, in relation to lesion morphology. To this end, we used a panel of established macrophage polarisation markers and inflammatory factors. Next, to investigate whether GM‐CSF and M‐CSF signals are crucial drivers of macrophage polarisation, their effects on macrophage differentiation and phenotypes were determined *in vitro*. In addition, we assessed the expression of GM‐CSF and M‐CSF in GCA lesions.

## Results

### Leucocyte infiltrates detected in different compartments of the arterial wall in GCA‐affected temporal arteries, GCA‐affected aortas and atherosclerotic aortas

Transmural inflammation was found in all GCA‐positive TABs, whereas no leucocyte infiltrates were found in the non‐inflamed control TABs (Supplementary figure 1a and b). GCA‐positive TABs presented with a high degree of intimal hyperplasia and luminal occlusion, whereas control TABs presented with no or minimal intimal hyperplasia. In the aortas from patients diagnosed with GCA, infiltrating leucocytes were found mainly in the adventitial and medial layers of the vessel wall (Supplementary figure 1c). The infiltrates in the media of the GCA aorta often formed a granulomatous rim around necrotic areas. This granulomatous infiltration pattern was not found in atherosclerotic aortas. In atherosclerotic aortas, however, adventitial infiltrates and massive intimal infiltrates surrounding plaques with minimal medial infiltration were found (Supplementary figure 1d).

### Distinct spatial distribution patterns of different macrophage phenotypes in GCA‐affected TABs

**Figure 1:**
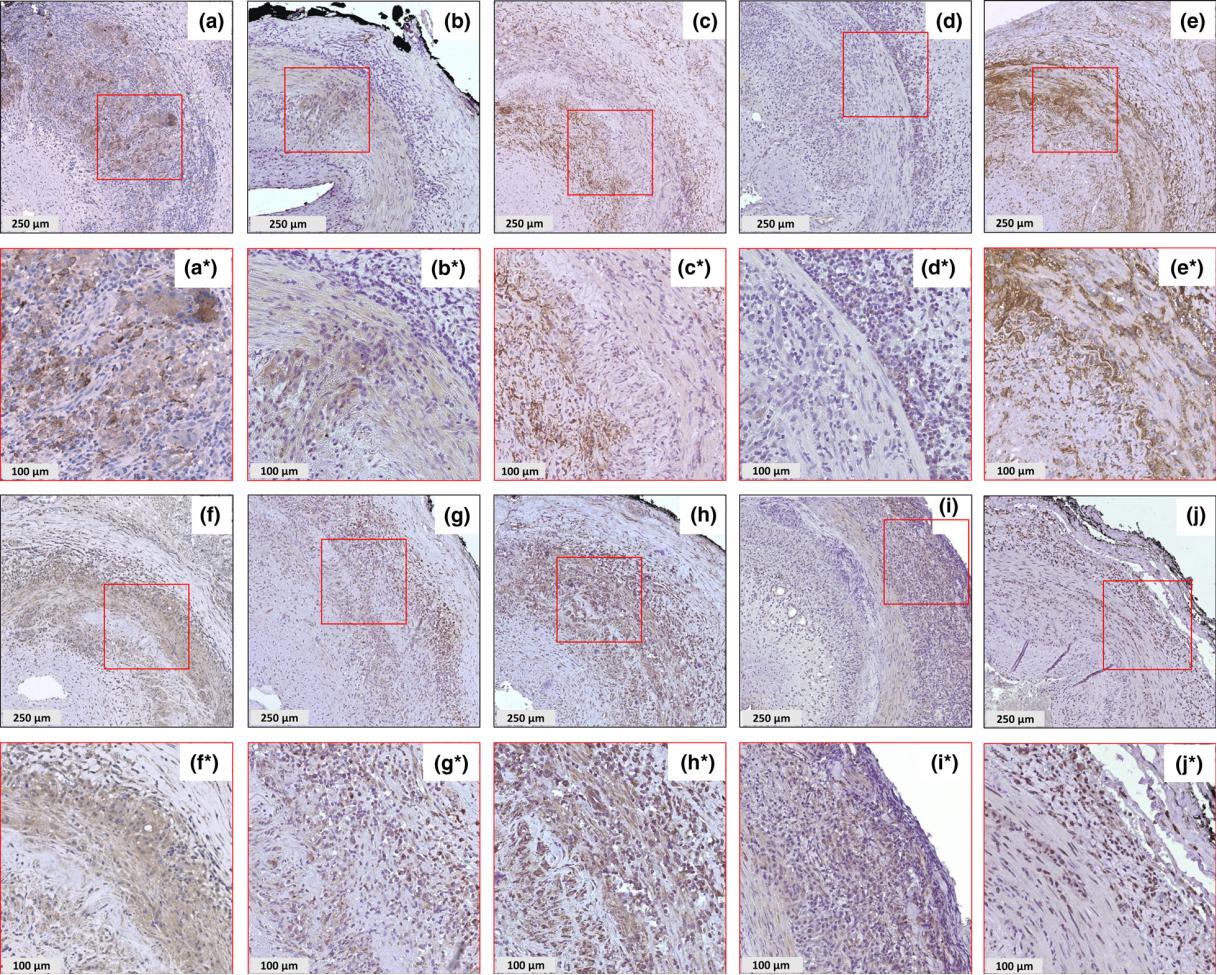
Expression of macrophage phenotypic markers, proinflammatory cytokines and matrixmetalloproteinases in GCA TABs. Single‐staining immunohistochemistry showed the expression of CD206 (a, a*), MMP‐9 (b, b*), FRβ (c, c*), MMP‐2 (d, d*), CD64 (e, e*), CD86 (f, f*), IL‐12 (g, g*), IL‐23 (h, h*),IL‐6 (i, i*) and IL‐1β (j, j*). Representative stainings are shown. Red box indicates zoomed region. Zoomed figures (*). GCA, giant cell arteritis; TAB, temporal artery biopsy.

In GCA TABs, markers of tissue remodelling macrophages were expressed in different layers of the vessel wall (Figure 1a–d and Supplementary figure 2 for all isotype controls). CD206 positivity was found mainly in the media, adventitia‐media and media‐intima borders (Figure 1a). Interestingly, MMP‐9 staining was also positive in CD206‐positive regions, suggesting that CD206^+^ macrophages express MMP‐9 (Figure 1b). In contrast, FRβ‐positive cells were mainly found in the adventitia and inner intima but rarely in the media (Figure 1c). Proinflammatory markers CD64, CD86, IL‐12, IL‐23, IL‐6 and IL‐1β (Figure 1e–j, respectively) were strongly expressed in all three layers of the vessel wall, most prominently in the adventitia. Additionally, MMP‐2 was detected mainly in the adventitia and the media (Figure 1d). This suggests that all macrophages in the vessel wall express CD64 and are capable of producing proinflammatory cytokines, but concomitantly express different tissue remodelling markers in specific compartments of the lesions.

Expression of CD64, FRβ, CD206 and MMP‐9 exclusively colocalised with PU.1 staining (Supplementary figure 3). PU.1, a transcription factor highly expressed in macrophages, was selected for double staining as it is found in cell nuclei, thereby facilitating visualisation of colocalisation with the cytoplasmic and membrane staining patterns of the other markers. Double staining of CD68 with PU.1 (Supplementary figure 3) showed that the vast majority of PU.1‐positive cells in TABs also expressed CD68, indicating that these cells are indeed macrophages. Although PU.1 can be expressed by mast cells, granulocytes, osteoclasts and Th9 cells, these cells are rarely found in GCA lesions while macrophages account for the majority of PU.1‐positive cells in GCA‐affected vessel walls. In contrast, some cells expressing the proinflammatory cytokines IL‐12 and IL‐23 stained negative for PU.1, indicating that besides macrophages, other infiltrating or tissue‐resident cells are also able to express these cytokines.

**Figure 2:**
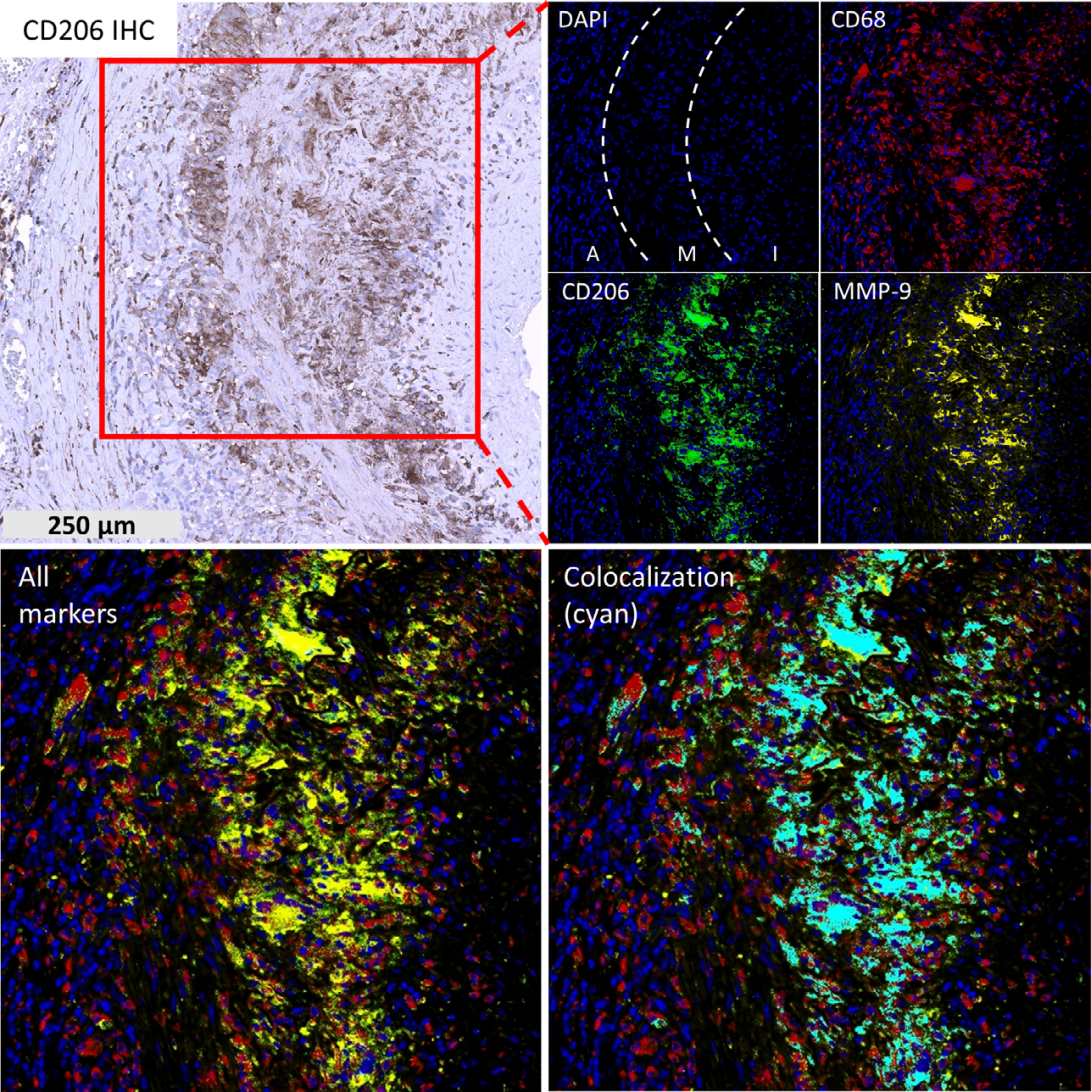
MMP‐9 is exclusively expressed by CD206^+^ macrophages in the media and media borders. Shown are the single‐staining immunohistochemistry of CD206 and the triple‐fluorescence staining of CD68 (red), CD206 (green) and MMP‐9 (yellow) on GCA TAB. Colocalisation of CD68, CD206 and MMP‐9 is shown in cyan. A, Adventitia; GCA, giant cell arteritis; I, Intima; M, Media; TAB, temporal artery biopsy.

**Figure 3:**
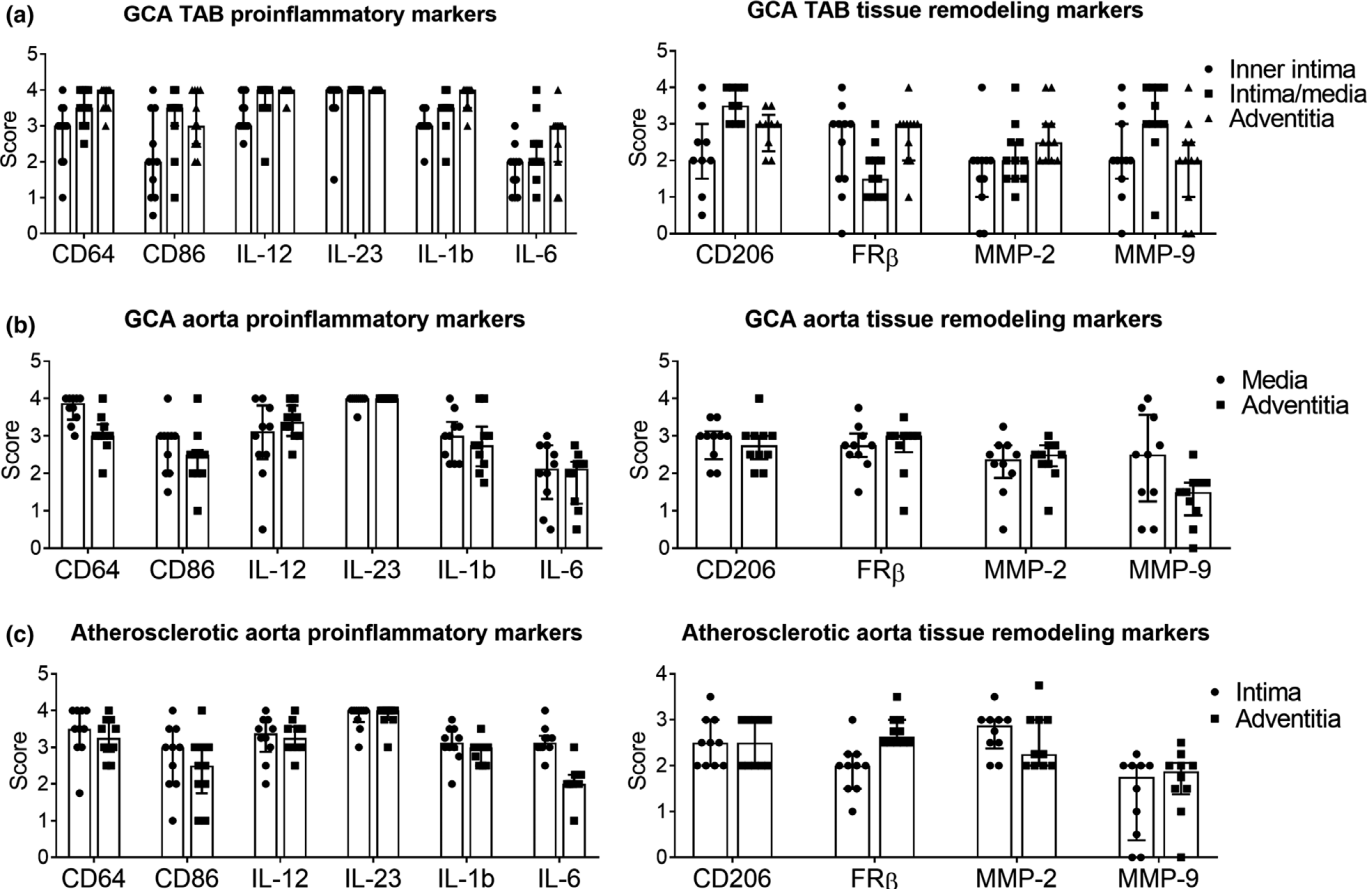
Localisation of proinflammatory and tissue remodelling markers in GCA TABs, GCA aortas and atherosclerotic aortas. Expression of surface markers, cytokines and matrix metalloproteinases (MMPs) in GCA‐affected TABs (*n* = 11, for CD206 *n* = 9), **(a)**, GCA‐affected aortas (*n* = 10), **(b)** and atherosclerotic aortas (*n* = 10), **(c)** was semiquantitatively scored. Data are presented as median with interquartile range. The intimal layer of GCA aortas and the medial layer of atherosclerotic aortas were not scored because of a lack of infiltrating cells. GCA, giant cell arteritis; MMP, matrix‐metalloproteinase; TAB, temporal artery.

Analysis of consecutive tissue stainings revealed a distinct distribution pattern of tissue remodelling markers in specific areas of the vessel (Supplementary figure 4a). More specifically, CD206^+^/MMP‐9^+^ macrophages were mainly found along the media and its borders, whereas FRβ^+^ macrophages were dominant in the inner intima and in the adventitia, areas adjacent to CD206^+^ macrophages. Triple‐fluorescence staining confirmed that only a subpopulation of CD68^+^ macrophages, located in the media and its borders, expressed CD206 and that MMP‐9 expression exclusively colocalised with these CD206^+^ macrophages (Figure 2). The latter would be in line with a tissue‐invasive role of CD206^+^/MMP9^+^ macrophages. Semiquantitative analysis of the tissue stainings further corroborated the distinct spatial distribution pattern of CD206^+^/MMP9^+^ and FRβ^+^ macrophages in the GCA vessel wall (Figure 3).

### Distribution pattern of macrophage phenotypes in GCA‐affected aortas and atherosclerotic aortas

All surface markers and cytokines were found to be abundantly expressed in both GCA and atherosclerotic aortas, albeit in different layers (Figure 3). The distinct pattern of different macrophage phenotypes found in GCA‐affected TABs was also found in the media of GCA‐affected aortas (Supplementary figure 4b). In the granulomatous rim, around sites of tissue necrosis, CD206^+^/MMP‐9^+^ macrophages were surrounded by FRβ^+^ macrophages. This distinct pattern of macrophage phenotypes, however, was not found in atherosclerotic aortas (Supplementary figure 4c). Macrophages around atherosclerotic plaques showed a mixed phenotype, with an overlapping expression of CD64, CD206 and FRβ without a distinct distribution pattern.

### GM‐CSF and M‐CSF contribute to macrophage phenotypic differences

As GM‐CSF and M‐CSF are known to influence macrophage phenotypes, we hypothesised that they play a key role in skewing macrophage phenotypes in GCA lesions. To test this hypothesis, we investigated the effects of GM‐CSF and M‐CSF on the phenotype of monocyte‐derived macrophages *in vitro*. GM‐MØs and M‐MØs from healthy donors and GCA patients were analysed for expression of CD64, CD86, CD206 and FRβ by flow cytometry. The culture supernatant was analysed for IL‐1β, IL‐6, IL‐12, IL‐23 and MMP‐9.

**Figure 4:**
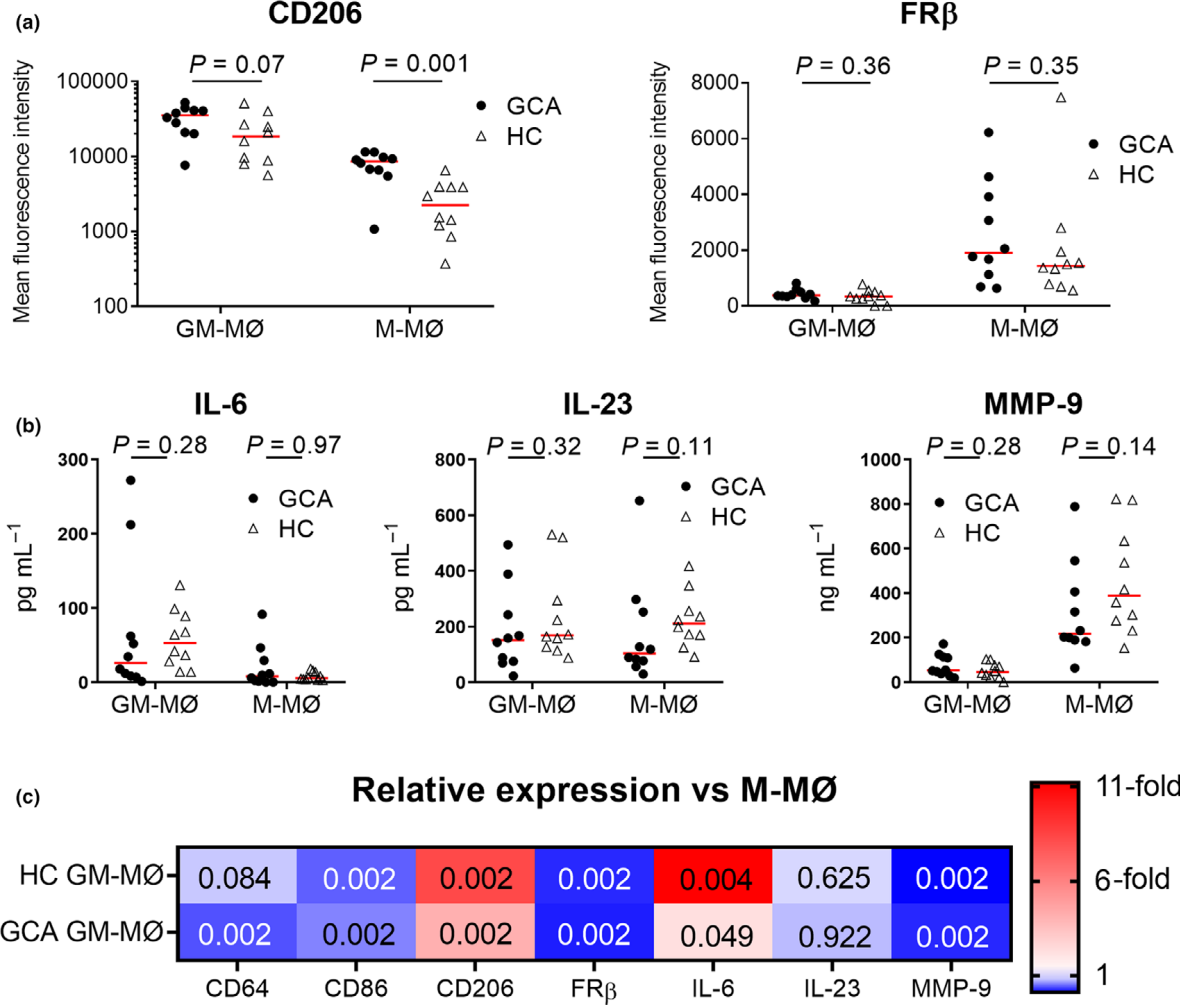
Macrophage surface marker expression and cytokine production depend on GM‐CSF and M‐CSF signals. Mean fluorescence intensity of CD206 and FRβ on GM‐CSF‐differentiated macrophages (GM‐MØs) and M‐CSF‐differentiated macrophages (M‐MØs) from GCA patients (*n* = 10) and healthy controls (*n* = 10) **(a)**. Luminex assay (normalised per 50 000 cells) of IL‐6, IL‐12 and MMP‐9 in culture supernatants of GM‐MØs and M‐MØs from GCA patients (*n* = 10) and healthy controls (*n* = 10) **(b)**. Heat map showing relative expression of the markers on GM‐MØs compared to M‐MØs **(c)**. GCA, giant cell arteritis.

CD206 expression was found to be significantly higher on GM‐MØs compared to M‐MØs (Figure 4a and c). FRβ, however, was expressed only on M‐MØs. Additionally, also CD64 and CD86 levels were higher on M‐MØs (Figure 4c). Compared to unstimulated monocyte subsets, both GM‐MØs and M‐MØs displayed an increased per‐cell expression of CD206, CD64 and CD86 (Supplementary figure 5). GM‐CSF signalling appeared to downregulate FRβ expression, as FRβ was expressed by monocytes but not by GM‐MØs.

Although clear phenotypic differences were observed by flow cytometry, only minor differences in cytokine production were found. While IL‐6, IL‐23 and MMP‐9 production were detected in supernatants (Figure 4b), IL‐12 and IL‐1β production were not (data not shown). IL‐6 levels were found to be significantly higher in the GM‐MØ supernatants than in the M‐MØ supernatants in both GCA and HCs (Figure 4b and c). For both GCA and HCs, MMP‐9 production was found to be significantly higher in M‐MØs than in GM‐MØs.

Additionally, the phenotypic differences observed in macrophages from GCA patients may, to some extent, already be present in circulating monocytes. By flow cytometry, differences in the expression of these markers on monocyte subsets, defined by CD14 and CD16 expression, were indeed observed (Supplementary figure 5). Interestingly, we found elevated expression of CD64 on classical and intermediate monocyte subsets from GCA patients compared to HCs. In contrast, FRβ expression on classical and intermediate monocytes from GCA patients was significantly lower compared to those from HCs. Although CD206 expression on monocyte subsets was not modulated in patients, CD206 expression was higher in GCA GM‐MØs and GCA M‐MØs than in their counterparts from HCs. Thus, circulating monocytes from GCA patients demonstrate a phenotype reminiscent of GM‐CSF‐stimulated macrophages.

### Local GM‐CSF and M‐CSF expression patterns may link to the distinct macrophage distribution pattern in GCA lesions

**Figure 5:**
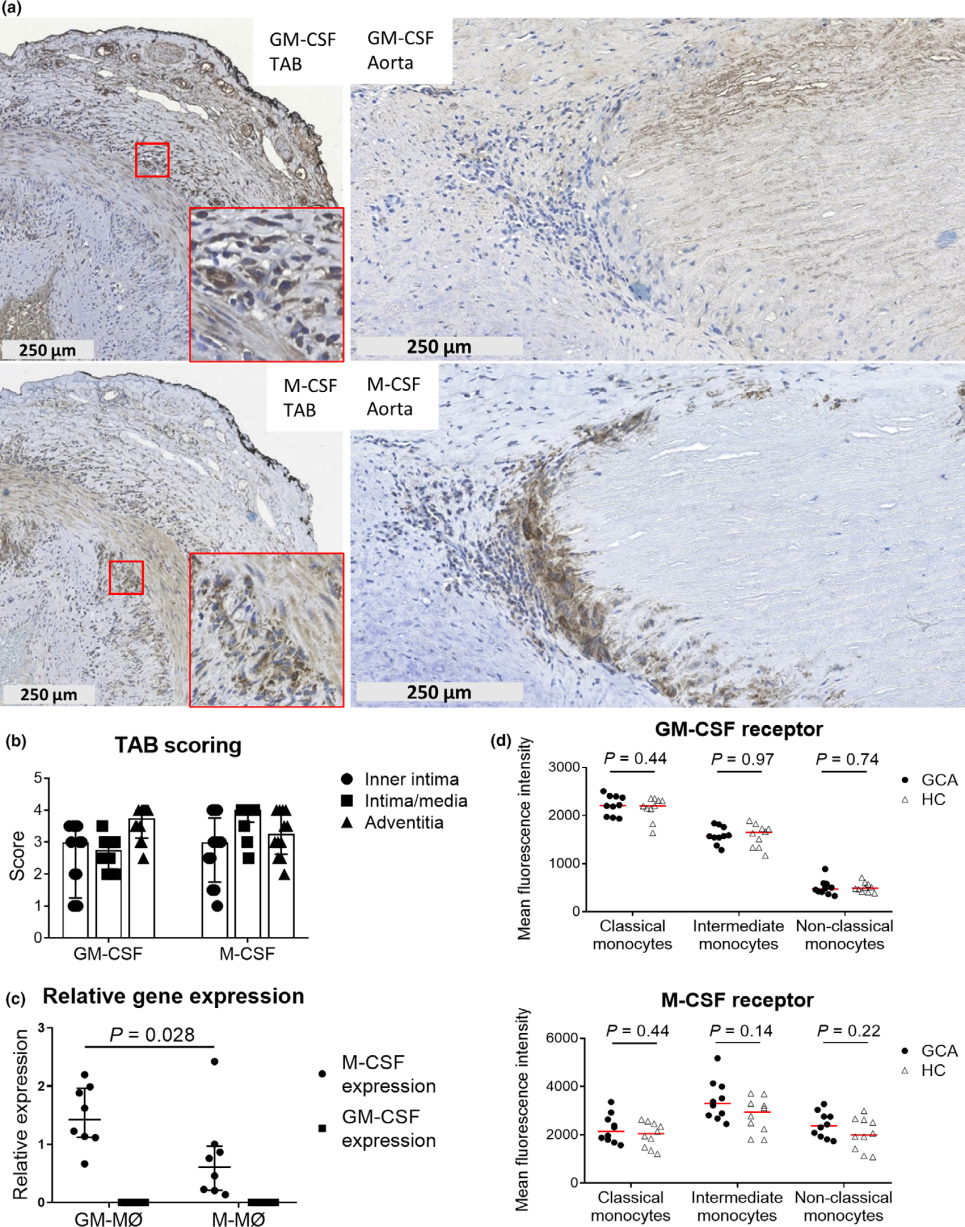
GM‐CSF and M‐CSF signalling in GCA tissues, macrophages and monocyte subsets. Tissue expression of GM‐CSF and M‐CSF in the temporal artery (TAB) and aorta biopsy tissues from GCA patients **(a)**. In the TABs, regions of interest (red) are magnified and shown in the lower right corner. In **(b)**, semiquantitative scores for GM‐CSF and M‐CSF in GCA TABs (*n* = 11) are displayed. The relative GM‐CSF and M‐CSF gene expression of healthy donor PBMC‐derived GM‐MØs and M‐MØs (*n* = 8 each, qPCR was performed in triplicates) normalised to β‐actin are shown in **(c)**. In **(d)**, the mean fluorescence intensity of the GM‐CSF receptor and the M‐CSF receptor in PBMC‐derived monocytes from healthy controls (HC) and GCA patients (*n* = 10 each) is shown. GCA, giant cell arteritis; GM‐MØ, GM‐CSF macrophages; M‐MØ, M‐CSF macrophages; TAB, temporal artery biopsy.

As we observed distinct effects of GM‐CSF and M‐CSF on macrophage surface marker expression, we performed IHC for GM‐CSF and M‐CSF on GCA‐affected TABs and aortas. These experiments revealed that GM‐CSF is dominantly expressed by infiltrating leucocytes and endothelial cells in the adventitial layer of GCA TABs (Figure 5a). In contrast, M‐CSF was found to be abundantly expressed at the site of the CD206^+^/MMP9^+^ macrophages at the intima‐media borders in TABs. These findings were substantiated by semiquantitative scoring showing the highest M‐CSF score in the media‐intima of TABs (Figure 5b). In the aorta, GM‐CSF was only weakly expressed in medial granulomas, whereas M‐CSF was highly expressed at the site of the CD206^+^ macrophages surrounding the necrotic areas (Figure 5a).

To assess the production of GM‐CSF and M‐CSF by skewed macrophages, we performed real‐time qPCR on total mRNA from GM‐MØs and M‐MØs (derived from HCs). We observed significantly higher expression of M‐CSF transcripts in GM‐MØs than in M‐MØs (Figure 5c). GM‐CSF transcripts, however, were not detected. This finding is in line with the tissue staining experiments, where M‐CSF was found expressed at the site of the CD206^+^ macrophages in the media and media borders, suggesting that these macrophages produce M‐CSF.

As CD206 expression was observed to be higher in GCA GM‐MØs and M‐MØs than in their HC counterparts, we reasoned that per‐cell expression of the GM‐CSF and M‐CSF receptors might be modulated in GCA monocytes. This, however, did not appear to be the case in peripheral blood monocyte subsets from HCs and GCA patients, in which no differences were found (Figure 5d).

Taken together, our data suggest that the expression pattern of GM‐CSF and M‐CSF in GCA lesions is different and may contribute to the spatial distribution of macrophage phenotypes in GCA lesions. M‐CSF produced by CD206^+^ macrophages is likely to prime adjacent macrophages to express FRβ.

### Macrophage FRβ positivity in the inner intima is associated with intimal hyperplasia

**Figure 6:**
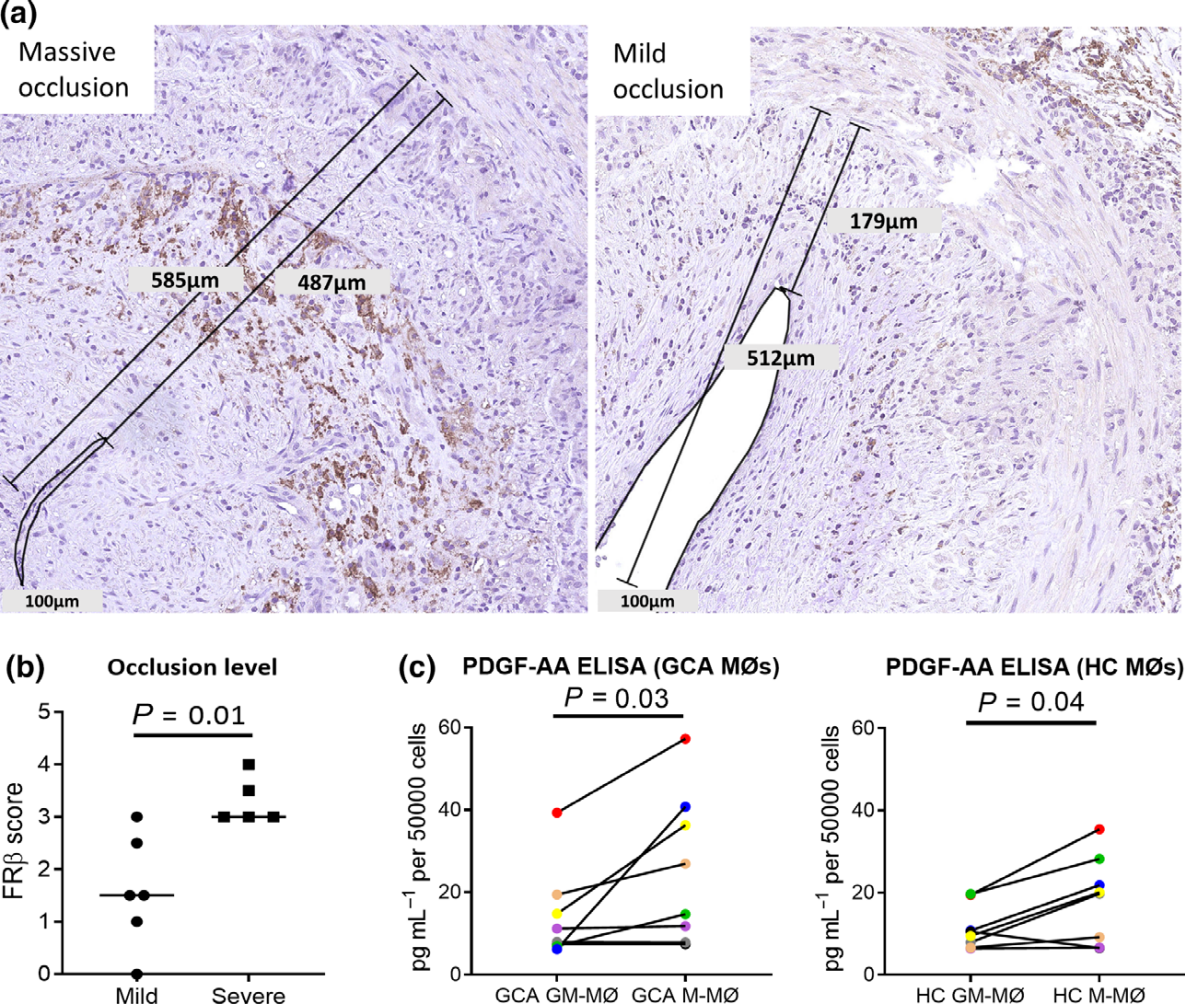
FRβ^+^ positivity in the inner intima is associated with high‐degree intimal hyperplasia. Classification of mildly and massively occluded TAB based on the thickness of intima **(a)**. The Mann–Whitney *U*‐test showed significantly higher expression of FRβ in the inner intima of TABs with massive intimal hyperplasia (mild occlusion *n* = 6; massive occlusion *n* = 5) **(b)**. The Wilcoxon signed‐rank test showed higher expression of PDGF‐AA in M‐MØ compared to GM‐MØ (*n* = 10 each, ELISA was performed in duplicates for each sample) **(c)**. GCA, giant cell arteritis; GM‐MØ, GM‐CSF macrophages; M‐MØ, M‐CSF macrophages; TAB, temporal artery biopsy.

FRβ positivity was found in the inner intima of the TAB, this is the region where intimal proliferation occurs. To determine whether the extent of FRβ positivity was associated with the severity of intimal hyperplasia, we divided luminal occlusion in GCA‐affected TABs into mild or massive occlusion based on the intimal thickness score (Figure 6a) and related this factor to the extent of FRβ positivity. Indeed, FRβ expression was higher in the inner intima region of TABs with massive intimal hyperplasia (Figure 6b).

Growth factors such as platelet‐derived growth factor (PDGF) could contribute to intimal hyperplasia. To investigate whether FRβ^+^ macrophages could be a source of PDGF, we performed macrophage differentiation and activation experiments followed by ELISA for PDGF‐AA in the culture supernatant. Our results showed that M‐MØs produced significantly higher concentrations of PDGF‐AA compared to GM‐MØs (Figure 6c), in both GCA and HC groups. Our data suggest that M‐MØs, resembling the FRβ^+^ macrophages indeed have the capacity to produce higher levels of a growth factor‐promoting intimal hyperplasia.

## Discussion

In this study, we revealed a distinct spatial distribution of macrophage phenotypes in GCA‐affected vessel walls and provide evidence that macrophage phenotypic heterogeneity is influenced by the growth factors GM‐CSF and M‐CSF. Moreover, distinct macrophage phenotypes were associated with tissue destruction and intimal hyperplasia. Although it has been suggested previously that macrophages have different functions in different compartments of the inflamed vessel wall in GCA,^17^ our study is the first to assign different macrophage phenotypes to defined regions of the vessel wall based on a broad selection of macrophage markers.

Our data demonstrate that macrophages in GCA‐affected TAB show a distinct expression pattern of surface markers depending on their location in the tissue. Macrophages with a proinflammatory phenotype, including expression of Th1‐ or Th17‐skewing cytokines, were detected throughout the vessel wall. CD206^+^/MMP‐9^+^ macrophages were mainly found in the media and media borders along the sites of lamina elastica degradation which is in line with a previous report that MMP‐9‐producing macrophages are located in the media borders.^8^, ^10^ Diversely, FRβ‐expressing macrophages were mainly found in regions adjacent to the CD206^+^ macrophages.

Our data suggest a role for GM‐CSF and M‐CSF in macrophage phenotypic heterogeneity in GCA lesions. Previously, CD206 and FRβ were found to be markers for GM‐CSF‐ and M‐CSF‐differentiated macrophages, respectively.^13^, ^14^ Our *in vitro* differentiation data confirmed that GM‐MØs indeed have high expression of CD206 and lack expression of FRβ. In contrast, FRβ expression was exclusively upregulated in M‐MØs. Additionally, GM‐CSF has been reported to diminish FRβ expression by macrophages even when differentiated in the presence of both GM‐CSF and M‐CSF.^14^ These findings suggest that CD206^+^ macrophages in the media and media borders are mainly primed by GM‐CSF, while FRβ^+^ macrophages adjacent to CD206^+^ macrophages are primed by M‐CSF. This also implies a gradient of GM‐CSF and M‐CSF production in different layers of the vessel wall that may be responsible for the distinct macrophage phenotype distribution observed. GM‐CSF expression was found highest in the adventitia and was mainly expressed by endothelial cells and infiltrating leucocytes, presumably activated T cells and B cells.^20^, ^21^ In contrast to GM‐CSF, M‐CSF expression was localised at the site of medial CD206^+^/MMP9^+^ macrophages. GM‐CSF can induce M‐CSF production, as previously demonstrated in monocytes^22^ and confirmed by our qPCR data (Figure 5c). Additionally, a recent study showed upregulated expression of GM‐CSF receptor in GCA‐affected temporal arteries.^23^ Furthermore, GM‐CSF in combination with IFNγ has been reported to significantly increase macrophage fusion into giant cells,^24^ the hallmark of GCA.^1^ Based on our immunohistochemical stainings, we found that multinucleated giant cells in GCA lesions are CD206^+^ and are located at the site of medial destruction.

We found significantly higher expression of FRβ in the inner intima in TABs with a higher degree of intimal hyperplasia. This finding suggests that FRβ macrophages may play a role in myofibroblast activation, migration and proliferation, leading to intimal hyperplasia. Indeed, improvement of pulmonary fibrosis was shown by depleting FRβ^+^ macrophages.^25^ FRβ expression has previously been reported in the adventitia of GCA TABs.^26^ Here, we showed that FRβ is also expressed in the inner intima. This discrepancy can be explained by differences in the degree of intimal hyperplasia in the TABs between studies. Mechanistically, FRβ^+^ M‐MØs in the inner intima may contribute to intimal hyperplasia by increased growth factor production such as PDGF‐AA.^27^ PDGF‐AA is expressed in GCA lesions and could contribute to myofibroblast activation, migration and proliferation.^28^, ^29^ Other growth factors such as FGFs, PDGF‐CC and PDGF‐DD could also potentially play a role in promoting intimal hyperplasia, although their relation to FRβ^+^ macrophages in GCA remains to be elucidated.

**Figure 7:**
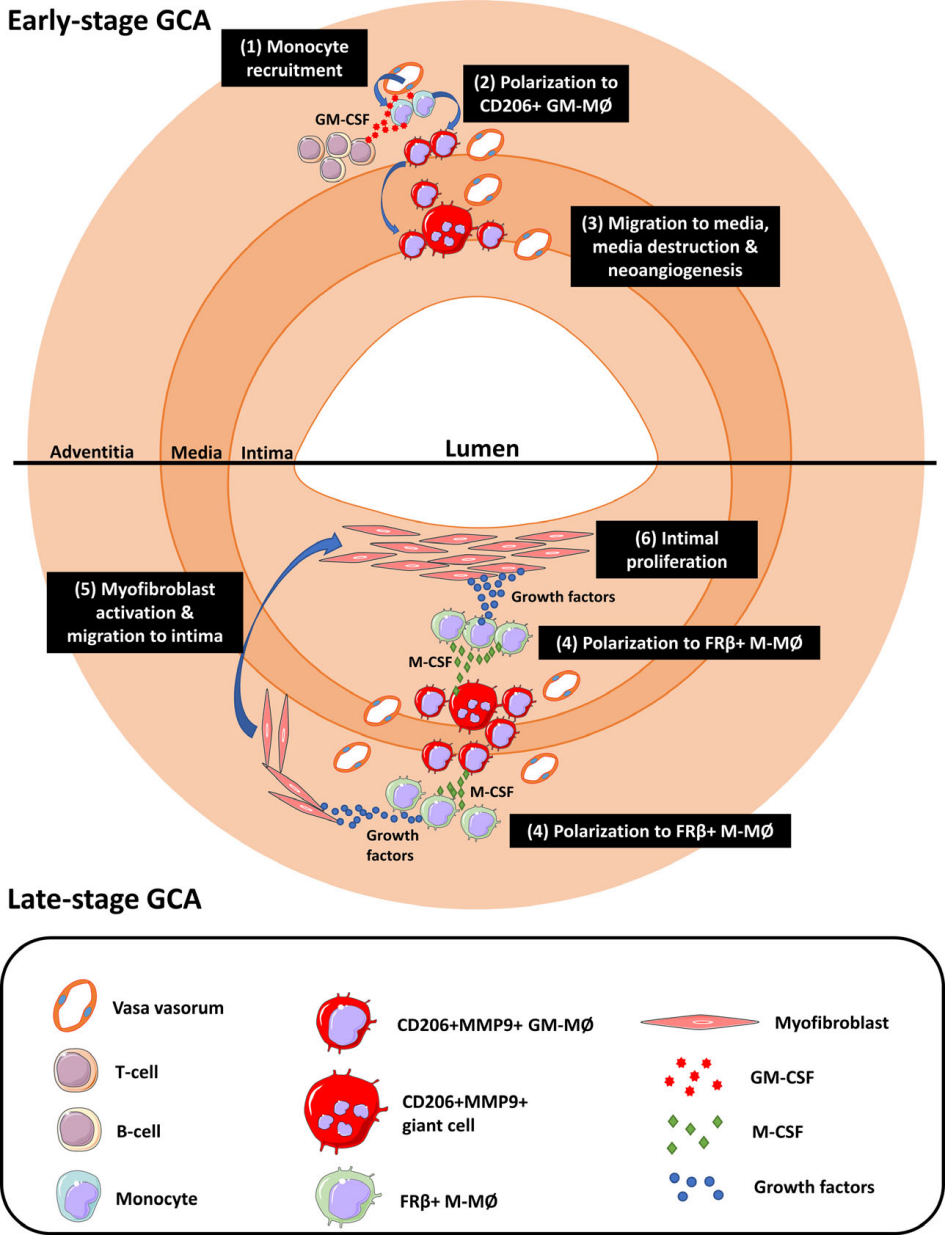
Model of step‐by‐step giant cell arteritis (GCA) pathogenesis in the temporal artery involving GM‐CSF and M‐CSF. The following steps occur in early‐stage GCA. (1) Monocytes enter the vessel wall. (2) Infiltrating monocytes are primed by GM‐CSF produced by T cells, B cells and endothelial cells, after which they differentiate into CD206^+^ GM‐MØs. (3) These CD206^+^ GM‐MØs then migrate to the media and media borders, exerting their tissue‐invasive, digestive and proangiogenic capabilities. In late‐stage GCA, the following steps occur. (4) CD206^+^ GM‐MØs release large amounts of M‐CSF, which in turn primes the macrophages surrounding them to express FRβ. (5) FRβ^+^ macrophages release high concentrations of growth factors that activate myofibroblasts, promoting their migration to the intima and (6) inducing their proliferation, which causes intimal hyperplasia and ultimately leads to luminal occlusion.

Macrophage heterogeneity in GCA lesion could be influenced by step‐wise GM‐CSF and M‐CSF signals. Recently, Watanabe *et al*.^30^ proposed two nonmutually exclusive pathways by which monocyte‐derived macrophages contribute to tissue injury and repair. In the first pathway, tissue‐infiltrating monocytes progressively differentiate from proinflammatory macrophages into proresolving macrophages depending on signals they encounter within the local microenvironment. In the second pathway, the proinflammatory macrophages disappear once the inflammatory trigger has been cleared. A second wave of monocytes then enters the tissue, which differentiates into proresolving macrophages in response to the environmental cues. Based on our data, we propose a potential pathogenic model of GCA involving sequential macrophage differentiation events (Figure 7). In this model, infiltrating monocytes are initially primed by GM‐CSF, after which they differentiate into CD206^+^ macrophages. These GM‐MØs then migrate to the media and media borders, exerting their tissue‐invasive, digestive and proangiogenic effects. Additionally, these CD206^+^ GM‐MØs release large amounts of M‐CSF, which in turn primes the macrophages surrounding them to express FRβ. These FRβ^+^ macrophages then boost myofibroblast migration and proliferation, possibly by higher production of growth factors (such as PDGF‐AA). This process eventually leads to luminal occlusion.

In contrast to our tissue staining results, MMP‐9 production was found to be higher in M‐MØ than in GM‐MØ. This result may be explained by the limitations of the *in vitro* differentiation model. Indeed, the GCA tissue environment is much more complex and enriched with a multitude of cytokines. Cytokines such as IFNγ, which is highly expressed in GCA lesions,^31^ could further modulate the expression of surface markers, cytokines and MMPs. Indeed, it has been reported that IFNγ synergises with GM‐CSF to increase MMP‐9, IL‐12 and IL‐1β production in macrophages.^32^, ^33^, ^34^

A distinct macrophage distribution pattern was also observed in GCA‐affected aortas but not in atherosclerotic aortas. In contrast to TABs, a distinct macrophage distribution pattern was observed only within the media of the GCA aortas. The variation in this distribution pattern between TABs and the aorta may be caused by differences in vessel wall size and anatomical build‐up, as aortas have thicker media with multiple lamina elastica layers. In contrast to GCA, but confirming previous reports, macrophages in atherosclerotic aortas were found mainly in the intima surrounding the atherosclerotic plaques.^35^ These macrophages showed overlapping CD64, CD206 and FRβ expression. In line with our findings of FRβ positivity around atherosclerotic plaques, reports have shown that partial M‐CSF depletion reduced atherogenesis^36^ and that M‐CSF‐activated gene signatures are dominant in early atherogenesis.^37^ We demonstrated that CD206 did not colocalise with FRβ^+^ macrophages in GCA, whereas concomitant CD206/FRβ expression was shown in atherosclerosis macrophages. The Th2 cytokine IL‐4, highly expressed in atherosclerotic lesions but to a limited extent in GCA lesions, can upregulate CD206 expression on FRβ macrophages.^31^, ^38^, ^39^, ^40^ Importantly, GM‐CSF was reported to be important in necrotic core formation in late‐stage atherogenesis.^41^ Overall, although macrophages are abundant in both diseases, the environmental cues governing macrophage phenotypes and functions are different. For GCA, we propose a sequential evolution of macrophage polarisation that is initially driven by GM‐CSF followed by M‐CSF signals, whereas the opposite sequence of events occurs in atherogenesis.

We observed that circulating monocytes and monocyte‐derived macrophages from GCA patients display a GM‐CSF signature compared to HCs. This finding was reflected by lower FRβ expression on monocytes and higher CD206 expression after differentiation into macrophages. This also implies that monocytes from GCA patients have a stronger response to GM‐CSF. However, we found no difference in GM‐CSF receptor expression between the groups. Additionally, our previous study showed no elevation of GM‐CSF in the serum of GCA patients,^42^ implying that other factors confer increased sensitivity of GCA monocytes to GM‐CSF.

The major strength of our study is the comprehensive analysis of multiple markers of inflammation and tissue remodelling, which allows the identification of distinct macrophage phenotypes. Our biopsy tissues were obtained from treatment‐naive patients to exclude the potential effects of GCs on macrophage phenotypes. Future studies should, however, address the impact of GCs on the skewing of lesional macrophage phenotypes. Finally, we also included atherosclerotic aortas for comparison and found that the roles of macrophages in the pathogenic processes leading to these two diseases are indeed different. We identified a possible role for GM‐CSF and M‐CSF in the local skewing of macrophage phenotypes in GCA and substantiated this finding with *in vitro* differentiation studies. We are aware that our *in vitro* model does not fully capture the events in the tissue, as a plethora of cytokines that can lead to further skewing and activation of macrophages were not explored. Future studies should focus on expanding the proposed model to incorporate additional activating cytokines and *in vivo* intervention models.

Our study may aid in expanding current GCA pathogenic models and identifying markers for targeted therapy. Currently, a GM‐CSF receptor‐blocking antibody, mavrilimumab (NCT03827018), is being evaluated in a phase 2 clinical trial for the treatment of GCA. Our findings add to the rationale for targeting the GM‐CSF receptor in this disease. Additionally, reduced inflammation was shown in a rheumatoid arthritis cartilage explant model with a CD64‐targeted immunotoxin.^43^ Although further studies are still needed, targeting CD206 might also prove to be useful in reducing tissue destruction while targeting FRβ might prevent luminal occlusion in GCA.

Taken together, vascular lesions of GCA patients display a distinct spatial distribution pattern of macrophage phenotypes associated with tissue destruction and intimal hyperplasia that are likely influenced by local expression of M‐CSF and GM‐CSF. These findings contribute to improved insights into the pathogenesis of GCA and lay the foundation for designing new macrophage‐targeted therapies and imaging tracers.

## Methods

The data supporting the findings of this study are available from the corresponding author on reasonable request.

### Patients

Eleven inflamed TAB tissue samples of histologically proven GCA collected before the start of GC treatment were studied (Table 1). The diagnosis of GCA was based on a positive (panartertic) TAB, based on a pathologist's assessment or a positive ^18^F‐fluorodeoxyglucose (FDG) positron emission tomography/computed tomography (PET‐CT) for GCA. In addition, non‐inflamed TAB tissue from patients who had (PET‐CT‐proven) GCA, isolated PMR patients, and individuals who had neither GCA nor PMR (*n* = 5 each) were included as controls. PMR was diagnosed by a positive PET‐CT for PMR. Clinical and laboratory data for these patients were collected as part of our prospective cohort study. The study was approved by the institutional review board of University Medical Center Groningen (METc2010/222). Written informed consent was obtained from all study participants. All procedures were in compliance with the Declaration of Helsinki.

**Table 1:** Clinical characteristics of patients and controls included in the tissue study and the *in vitro* study

	GCA‐positive TAB	GCA aorta	Artherosclerotic aorta	GCA PBMCs	Healthy control PBMCs
*N*	11	10	10	10	10
Age (median; years)	74	66	65	72	72
Sex (% female)	70	70	50	70	70
Fulfilled ACR criteria (yes/no)	11/0	NA	NA	8/2	NA
Claudication (yes/no)	9/2	NA	NA	4/6	NA
Visual ischaemia (yes/no)	4/7	NA	NA	1/9	NA
PMR clinic (yes/no)	1/10	NA	NA	2/8	NA
CRP (mg L^−1^; median)	66	7	10	38	1.5
ESR (mm h^−1^; median)	83	13	15	73	9

ACR, American College of Rheumatology; CRP, C‐reactive protein; ESR, erythrocyte sedimentation rate; GCA, giant cell arteritis; PBMCs, peripheral blood mononuclear cells; PMR, polymyalgia rheumatica; TAB, temporal artery biopsy.

Aorta tissues from GCA patients (*n* = 10) and age‐matched atherosclerotic controls (*n* = 10) were retrospectively obtained after aortic aneurysm surgery (Supplementary table 1). None of the patients used GCs at the time of surgery. GCA was diagnosed after examination of the aortic tissue by pathologists. The patients' clinical and laboratory data at the time of surgery were extracted from medical records. Consent from the Internal Review Board and written patient consent were not required under Dutch law for human medical research (WMO) since the tissue was obtained during necessary surgery. The patients were informed about the study and agreed that the obtained medical data could be used for research purposes in accordance with privacy rules.

Frozen peripheral blood mononuclear cells (PBMCs) from treatment‐naive GCA patients (*n* = 10) and age‐ and sex‐matched healthy controls (HCs, *n* = 10) participating in the prospective cohort study were used for *in vitro* studies (Table 1). Additionally, for these patients, the GCA diagnosis was confirmed by TAB and/or PET‐CT. HCs were screened by health assessment questionnaires, physical examination and laboratory tests for past and actual morbidities and excluded when they were not healthy according to the adapted Senieur criteria.^44^

### Immunohistochemistry (IHC)

Formalin‐fixed, paraffin‐embedded tissues were cut into sections of 3 µm. The sections were deparaffinised and rehydrated, followed by antigen retrieval in a 95°C water bath (for buffers, see Supplementary table 1). For single staining, tissues were incubated with primary anti‐human antibodies (Supplementary table 1), followed by endogenous peroxidase blocking. The tissues were subsequently incubated with secondary antibodies, 3‐amino‐9‐ethylcarbazole (DAKO, Glostrup, Denmark) for peroxidase activity detection, and finally haematoxylin (MERCK, Kenilworth, NJ, USA) as a counterstain. Matching isotype controls were also included. For double staining with the macrophage transcription factor PU.1, tissues were simultaneously incubated with two primary antibodies (Supplementary table 1). A MultiVision alkaline phosphatase and horseradish peroxidase double‐staining kit (Thermo Fisher Scientific, Waltham, MA, USA) was used. Reactive tonsil tissue was used as a positive control tissue except for the detection of GM‐CSF where spleen tissue was used. All slides were scanned using a Nanozoomer Digital Pathology Scanner (NDP Scan U 10074‐01; Hamamatsu Photonics K.K., Shizuoka, Japan).

Giant cell arteritis‐positive TABs, GCA‐positive aortas and atherosclerotic aortas were semiquantitatively scored on a five‐point scale (0–4): 0 = no positive cells, 1 = occasional positive cells (0–1% estimated positive), 2 = small numbers of positive cells (> 1–20%), 3 = moderate numbers of positive cells (> 20–50%), 4 = large numbers of positive cells (more than 50%). An average score was calculated from assessments by two independent investigators. Tissues were scored in representative areas that contained infiltrating cells, as GCA can contain skip lesions.^45^

### Triple‐fluorescence multispectral imaging

Paraffin sections were deparaffinised and rehydrated, followed by antigen retrieval in Tris‐EDTA buffer (pH 9) in a 95°C water bath for 45 min. Tissues were incubated for 5 min with TrueBlack Lipofuschin autofluorescence blocker (Biotium, Fremont, CA, USA) in the dark at room temperature. Next, tissues were incubated with a cocktail of primary antibodies (Supplementary table 2). Subsequently, they were incubated with a cocktail of secondary antibodies, followed by incubation with a cocktail of tertiary antibodies tagged with fluorescence labels. Afterwards, the tissues were incubated with DAPI for 5 min as counterstain and sealed. Image cubes were captured at a magnification of 20× using Nuance Multispectral Imaging System 3.0.1 (PerkinElmer, Waltham, MA, USA) using NuanceFX 3.0.1 software (PerkinElmer). All filters available in the system were utilised to acquire the image cube with multiple wavelength acquisition (440:460 for DAPI, 490:530 for Alexa 488, 570:600 for Alexa 568, 710:720 for Alexa 647). Spectral unmixing was performed with spectral libraries of each fluorophore assigned different colours (DAPI = blue, Alexa 488 = green, Alexa 568 = red, Alexa 647 = yellow), subtracting the background autofluorescence. Colocalisation of the three fluorophores was analysed and assigned the colour cyan.

### Monocytes and monocyte‐derived macrophages *in vitro*

Giant cell arteritis and HC monocytes were isolated from thawed PBMCs by negative selection using the EasySep monocyte enrichment kit (Stemcell, Vancouver, BC, Canada), which does not deplete CD16^+^ monocytes. Isolated monocytes were analysed by flow cytometry or cultured for 7 days in DMEM containing 2 mm glutamine, 60 µg mL^−1^ penicillin–streptomycin and 10% FCS in the presence of 100 ng mL^−1^ GM‐CSF (Peprotech, Rocky Hill, CT, USA) to generate GM‐MØs or 100 ng mL^−1^ M‐CSF (Peprotech) to generate M‐MØs. The medium was replaced on the second and fourth day. On day 7, after collecting the supernatants for Luminex assay, monocyte‐derived macrophages were harvested using citrate saline (135 mm potassium chloride, 15 mm sodium citrate and 1 mm EDTA) for 15 min at 37°C. For macrophage activation experiments, monocytes were cultured for 7 days in the same medium in the presence of GM‐CSF/M‐CSF. The medium was replaced on the third and fifth days. LPS (100 ng mL^−1^) (Sigma‐Aldrich, St. Louis, MO, USA) was added to the culture during the fifth day medium change. On day 7, supernatants were collected for ELISA.

### Flow cytometry

Phenotyping of monocytes and monocyte‐derived macrophages was performed by flow cytometry using fluorochrome‐conjugated monoclonal antibodies specific for HLA‐DR (FITC, BD Biosciences Franklin Lakes, NJ, USA), CD14 (Pacific Orange, Thermo Fisher Scientific), CD16 (BUV737, BD), CD64 (APC‐Cy7, Biolegend, San Diego, CA, USA), CD86 (BV711, BD), CD206 (PE‐Cy7, Biolegend) and FRβ (APC, Biolegend). The expression of the GM‐CSF receptor (BV650, BD) and the M‐CSF receptor (PE‐Cy7, Biolegend) on monocyte subsets was analysed by a separate flow cytometry panel (including the aforementioned CD14, CD16 and HLA‐DR antibodies). Cells were measured on LSR‐II (BD) flow cytometer. For comparison of the mean fluorescence intensity between experiments, the LSR‐II flow cytometer was calibrated for each run using FACSDiva CS&T research beads (BD). Data were analysed using Kaluza software (BD). Monocytes and macrophages were gated by FSC/SSC, doublets were excluded, and dead cells were excluded using Zombie dye (Biolegend). To exclude contaminating lymphocytes in the monocyte gate, cells negative for both HLA‐DR and CD14 were gated out. Monocyte subsets were gated based on CD14 and CD16 expression.^6^ Gating strategy plots are available as Supplementary figure 6.

### Luminex assay and ELISA

Supernatants from the GM‐MØ and M‐MØ cultures (non‐activated and activated) were stored at −20°C until further use. In supernatants of non‐activated cultures, levels of IL‐1β, IL‐6, IL‐10, IL‐12, IL‐23 and MMP‐9 were measured with Human premix Magnetic Luminex screening assay kits (R&D Systems, Abingdon, UK) according to the manufacturer's instructions and read on a Luminex Magpix instrument (Luminex, Austin, TX, USA). Data were analysed with xPONENT 4.2 software (Luminex). PDGF‐AA concentrations in the supernatant of activated cultures were measured with the Human PDGF‐AA Duo set ELISA (DY‐221, R&D Systems) according to the manufacturer's instruction. For calculation and graphing purposes of the PDGF data, data points below the detection limit of 3.5 pg mL^−1^ were included and assigned the value of 1.75 pg mL^−1^. Supernatant levels were corrected for the macrophage cell count at the time of harvesting and are expressed as ng mL^−1^ per 50 000 cells.

### RNA extraction and qPCR

Total RNA was extracted from healthy donor‐derived GM‐MØs and M‐MØs using the RNeasy® Mini Kit (Qiagen, Hilden, Germany). Total RNA was reverse transcribed with SuperScript III reverse transcriptase (Invitrogen, Carlsbad, CA, USA) with random hexamers (Promega, Madison, WI, USA). Real‐time qPCR was conducted with a ViiA™ 7 Real‐Time PCR System with TaqMan™ probes (Thermo Fisher) targeting M‐CSF (CSF1, Hs00174164_m1) and GM‐CSF (CSF2, Hs00929873_m1). Amplification plots were analysed with QuantStudio™ Real‐Time PCR software v1.3 (Applied Biosystems, Foster City, CA, USA). Relative gene expression was normalised to β‐actin (ACTB, Hs99999903_m1) as an internal control.

### Statistics

To analyse the differences between HC and GCA, GM‐MØ and M‐MØ in the *in vitro* study, nonparametric Mann–Whitney *U*‐tests (two‐tailed) were used. Differences in FRβ expression scores for the inner intima between patients with low and high vessel occlusion scores were also assessed by Mann–Whitney *U*‐tests. Differences between the results of GM‐MØ and M‐MØ raised from the same donor were analysed with the paired Wilcoxon signed‐rank test.

## Conflict of interest

The authors declare no conflict of interest. AMHB was a consultant for Grünenthal Gmbh until 2017. EB and KvdG as employees of the UMCG received speaker/consulting fees from Roche which were paid to the UMCG. PH, WHA and EB have received funding from the European Union's Horizon 2020 research and innovation programme under grant agreement 668036.

## Author contributions

**William F Jiemy:** Conceptualization; Data curation; Formal analysis; Investigation; Methodology; Project administration; Validation; Visualization; Writing‐original draft; Writing‐review & editing. **Yannick van Sleen:** Conceptualization; Data curation; Formal analysis; Investigation; Methodology; Project administration; Validation; Visualization; Writing‐original draft; Writing‐review & editing. **Kornelis SM van der Geest:** Data curation; Project administration; Resources; Software; Supervision; Validation; Writing‐review & editing. **Hilde A ten Berge:** Data curation; Formal analysis; Investigation; Visualization; Writing‐review & editing. **Wayel H Abdulahad:** Formal analysis; Investigation; Supervision; Validation; Writing‐review & editing. **Maria Sandovici:** Supervision; Validation; Writing‐review & editing. **Annemieke MH Boots:** Conceptualization; Funding acquisition; Methodology; Resources; Supervision; Validation; Writing‐review & editing. **Peter Heeringa:** Conceptualization; Funding acquisition; Methodology; Project administration; Resources; Supervision; Validation; Writing‐review & editing. **Elisabeth Brouwer:** Conceptualization; Funding acquisition; Methodology; Project administration; Resources; Supervision; Validation; Writing‐review & editing.
