# Supplementary material for: Distinct macrophage phenotypes skewed by local granulocyte macrophage colony‐stimulating factor (GM‐CSF) and macrophage colony‐stimulating factor (M‐CSF) are associated with tissue destruction and intimal hyperplasia in giant cell arteritis
Source: Clin Transl Immunology. 2020 Aug 27;9(9):e1164. doi: 10.1002/cti2.1164 (PMC7453134; doi:10.1002/cti2.1164)
Supplement: Supplementary file 1 [file CTI2-9-e1164-s001.pdf]

Supplementary table 1. List of antibodies.

Single Staining								
**Target**	**Antigen retrieval**	**Manufacturer & code**	**Host**	**Isotype**	**Dilution**	**Secondary antibody**	**Manufacturer & Code**	**Dilution**
**IL-6**	pH 6	Santa Cruz, Dallas, TX, USA, SC-130326	Mouse	IgG2b	1:100	Rabbit α-mouse Ig-HRP	DAKO, Glostrup, Denmark, P260	1:50
**IL-1β**	pH 9	Abcam, Cambridge, UK Ab156791	Mouse	IgG1	1:100	Envision α-mouse polymer-HRP	DAKO, K4006	Undiluted
**IL-12p35**	pH 6	Atlas Antibodies, Bromma, Sweden, HPA001886	Rabbit	Polyclonal (IgG)	1:25	Envision α-rabbit polymer-HRP	DAKO, K4003	Undiluted
**IL-23p19**	pH 6	Abcam, Ab45420	Rabbit	Polyclonal (IgG)	1:100	Goat α-rabbit Ig-HRP	DAKO, P448	1:50
**CD206**	pH 9	R&D Systems, Abingdon, UK, MAB25341	Mouse	IgG2b	1:50	Envision α-mouse polymer-HRP	DAKO, K4006	Undiluted
**CD64**	pH 9	Abcam, Ab140779	Mouse	IgG1	1:150	Rabbit α-mouse Ig-HRP	DAKO, P260	1:50
**MMP-2***	pH 6	Abcam, Ab86607	Mouse	IgG2a	1:500	Rabbit α-mouse Ig-HRP	DAKO, P260	1:50
**MMP-9***	pH 9	Abcam, Ab58803	Mouse	IgG1	1:200	Rabbit α-mouse Ig-HRP	DAKO, P260	1:50
**FR-β**	pH 9	Origene, Rockville, MD, USA, TA808017	Mouse	IgG1	1:150	Rabbit α-mouse Ig-HRP	DAKO, P260	1:50
**M-CSF**	pH 9	Abcam, Ab52864	Rabbit	IgG	1:100	Goat α-rabbit Ig-HRP	DAKO, P448	1:50
**CD86**	pH 9	Abcam, Ab234000	Mouse	IgG1	1: 50	Rabbit α-mouse Ig-HRP	DAKO, P260	1:50
**GM-CSF**	pH 6	Abcam, Ab9741	Rabbit	Polyclonal (IgG)	1:250	Envision α-rabbit polymer-HRP	DAKO, K4003	Undiluted

Double Staining							
**Target**	**Antigen retrieval**	**Manufacturer & code**	**Host & Isotype**	**Dilution**	**Secondary antibody**	**Manufacturer & Code**	**Dilution**
**CD64**	pH 9	Abcam, Ab140779	Mouse, IgG1	1:150	MultiVision polymer cocktail	Thermo Fisher, Waltham, MA, USA, TL-012-MARH	Undiluted
**PU.1**		Abcam, Ab76543	Rabbit monoclonal IgG	1:150			
**CD206**	pH 9	R&D Systems, MAB25341	Mouse, IgG2b	1:40	MultiVision polymer cocktail	Thermo Fisher, TL-012-MARH	Undiluted
**PU.1**		Abcam, Ab76543	Rabbit monoclonal IgG	1:150			
**FR-β**	pH 9	Origene, TA808017	Mouse, IgG1	1:200	MultiVision polymer cocktail	Thermo Fisher, TL-012-MARH	Undiluted
**PU.1**		Abcam, Ab76543	Rabbit monoclonal IgG	1:150			
**MMP-9**	pH 9	Abcam, Ab58803	Mouse, IgG1	1:200	MultiVision polymer cocktail	Thermo Fisher, TL-012-MARH	Undiluted
**PU.1**		Abcam, Ab76543	Rabbit monoclonal IgG	1:150			
**IL-12**	pH 9	Atlas Antibodies, HPA001886	Rabbit polyclonal IgG	1:50	MultiVision polymer cocktail	Thermo Fisher, TL-012-MARH	Undiluted
**PU.1**		Biolegend, San Diego, CA, USA, 658012	Mouse, IgG1	1:20			
**IL-23**	pH 9	Abcam, Ab45420	Rabbit polyclonal IgG	1:150	MultiVision polymer cocktail	Thermo Fisher, TL-012-MARH	Undiluted
**PU.1**		Biolegend, 658012	Mouse, IgG1	1:20			

*: Antibodies against MMPs detect both pro-MMPs and active MMPs

Supplementary table 2. Triple fluorescence staining panel.

	Targets			
	**CD206 (R&D Systems, MAB25341)**	**MMP-9 (abcam, Ab58803)**	**CD68 (DAKO, M0876)**	**Nucleus**
**Isotype (primary ab)**	Mouse IgG2b 1:20	Mouse IgG1 1:150	Mouse IgG3 1:50	
**Secondary ab**	Rat anti-mouse IgG2b (Biolegend, RMG2b-1) 1:20	Rabbit anti-mouse IgG1 (Novus Biologicals, Littleton, CO, USA, NBP1-72793) 1:50	Goat anti-mouse IgG3 (Southern Bioteh, Birmingham, AL, USA, 1101-01) 1:75	
**Tertiary ab**	Donkey anti-rat IgG (abcam, ab150153) 1:40	Donkey anti-rabbit IgG (abcam, ab150075) 1:50	Donkey anti-goat IgG (abcam, ab175704) 1:75	
**Conjugate/dye**	AF488	AF647	AF568	DAPI
**Longpass filter channel**	515LP	610LP	590LP	475LP

*: Antibodies against MMPs detect both pro-MMPs and active MMPs

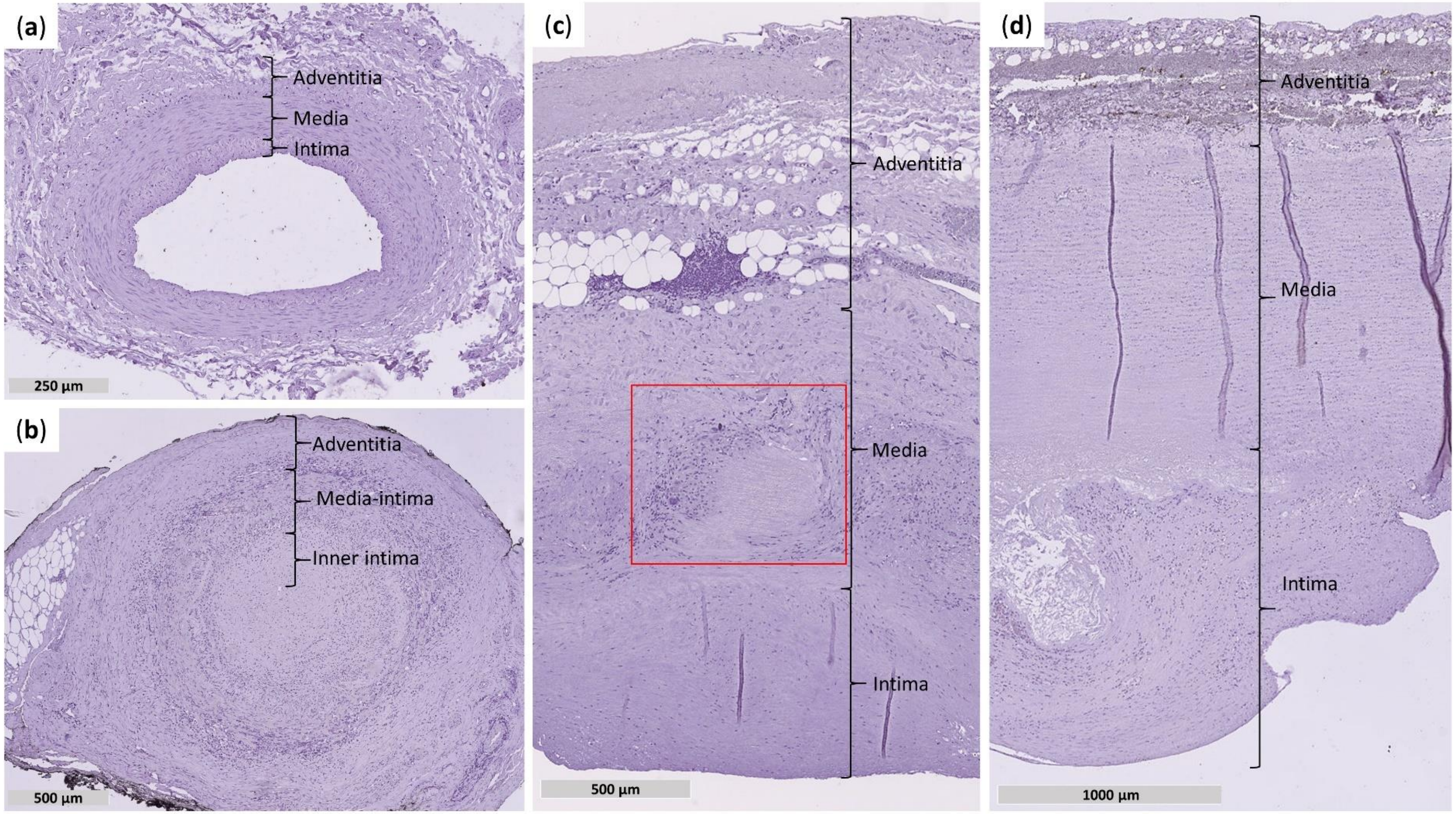

Supplementary figure 1. Tissue topology of the healthy and unhealthy vessel wall. Representative hematoxylin staining of a GCA-negative temporal artery biopsy (a), GCA-positive temporal artery biopsy (b), GCA-positive aorta (c) and atherosclerotic aorta (d). Infiltrating leukocytes can be found in all three layers of GCA-positive TABs, whereas no infiltrates were found in GCA-negative TABs. In GCA-positive aortas, infiltrating leukocytes localized mainly in the adventitia and media. In contrast, atherosclerotic aortas showed massive intimal infiltration with minimal infiltrates in the media. The red box shows a necrotizing granuloma with a leukocyte rim present in GCA-affected aortas but not in atherosclerotic aortas. GCA: giant cell arteritis, TAB: temporal artery biopsy.

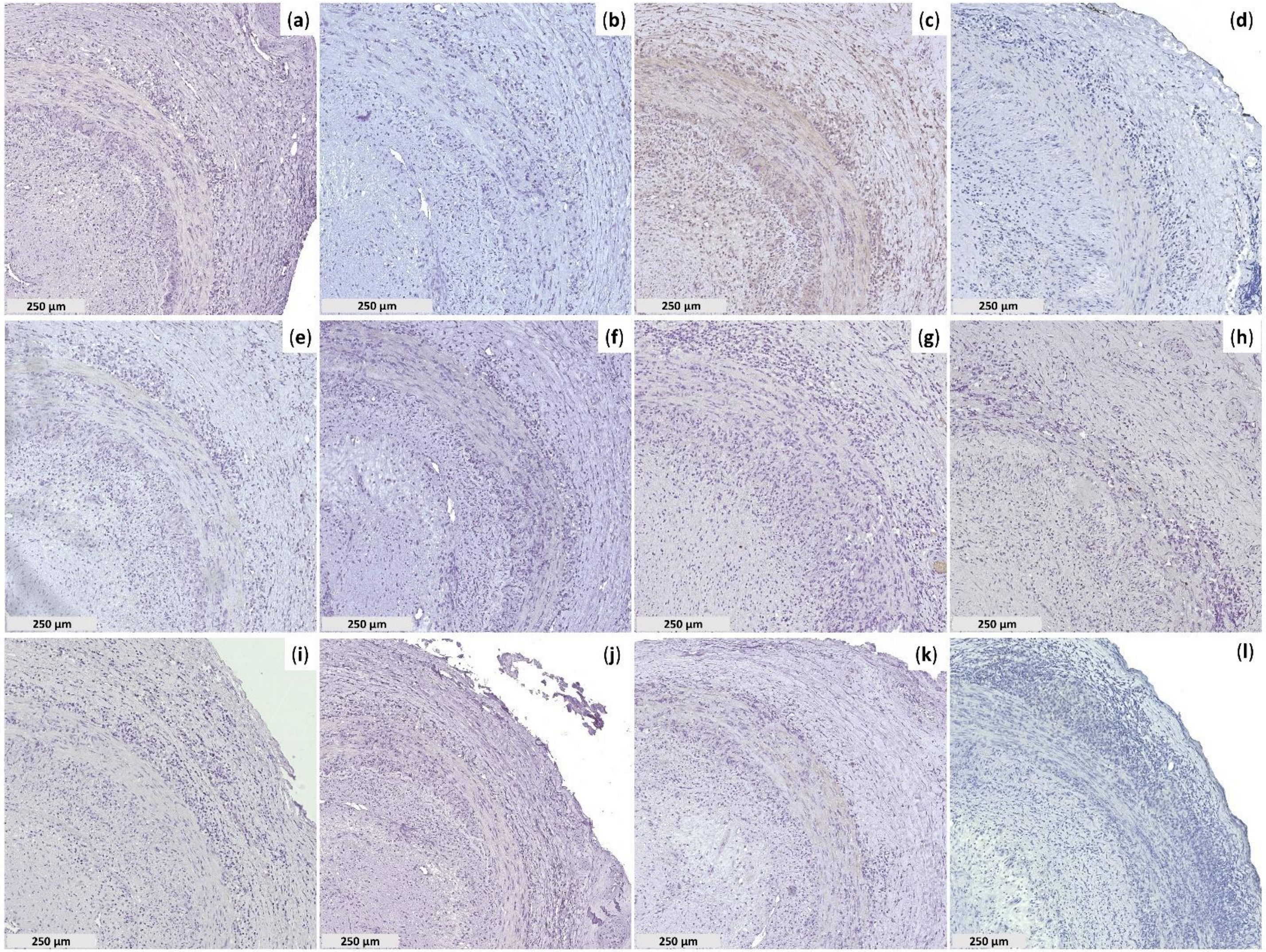

Supplementary figure 2. Isotype control for CD64 (a), CD206 (b), FRβ (c), CD86 (d), IL-12 (e), IL-23 (f), IL-1β (g), IL-6 (h), MMP-2 (i), MMP-9 (j), GM-CSF (k) and M-CSF (l).

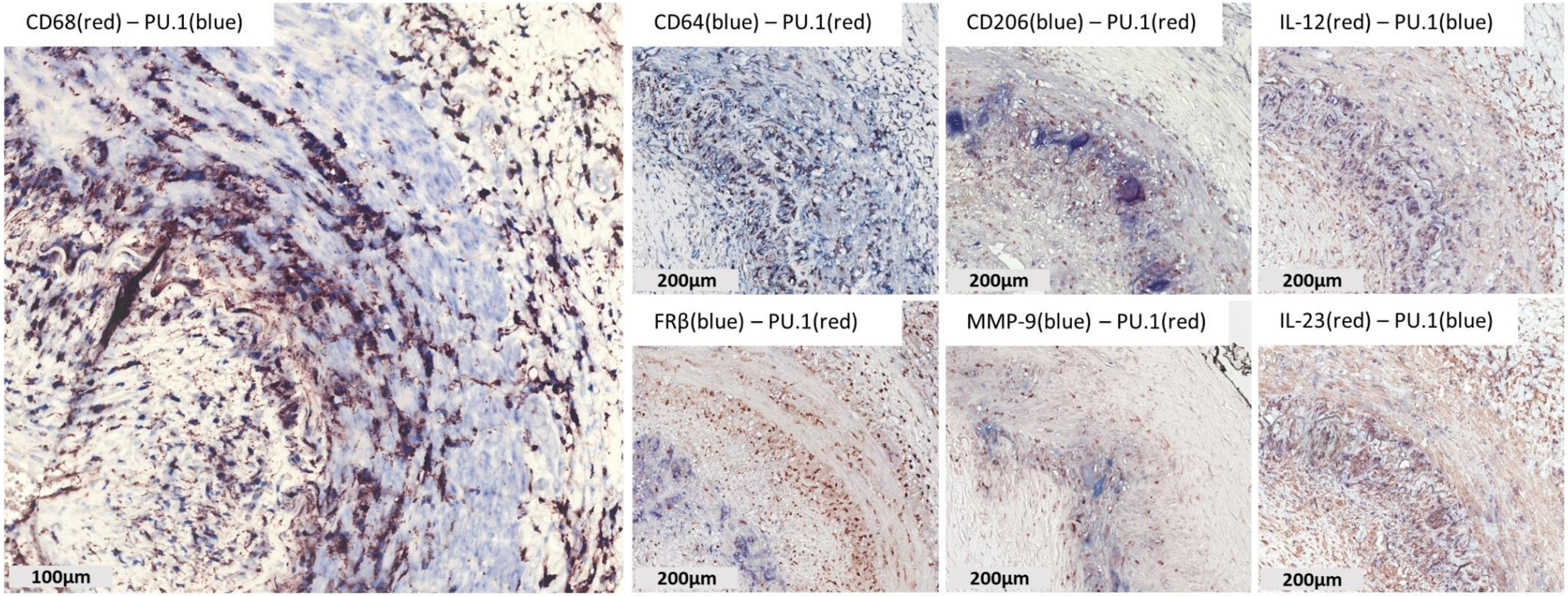

Supplementary figure 3. CD64, CD206, FRβ, IL-12, IL-23 and MMP-9 are expressed by macrophages. Shown are double staining of the macrophage phenotypic markers, proinflammatory cytokines and MMP-9 with macrophage transcription factor PU.1. Double staining of PU.1 and CD68 confirmed that PU.1 is a reliable macrophage marker for GCA TAB. GCA: giant cell arteritis, TAB: temporal artery biopsy, MMP: matrix-metalloproteinase.

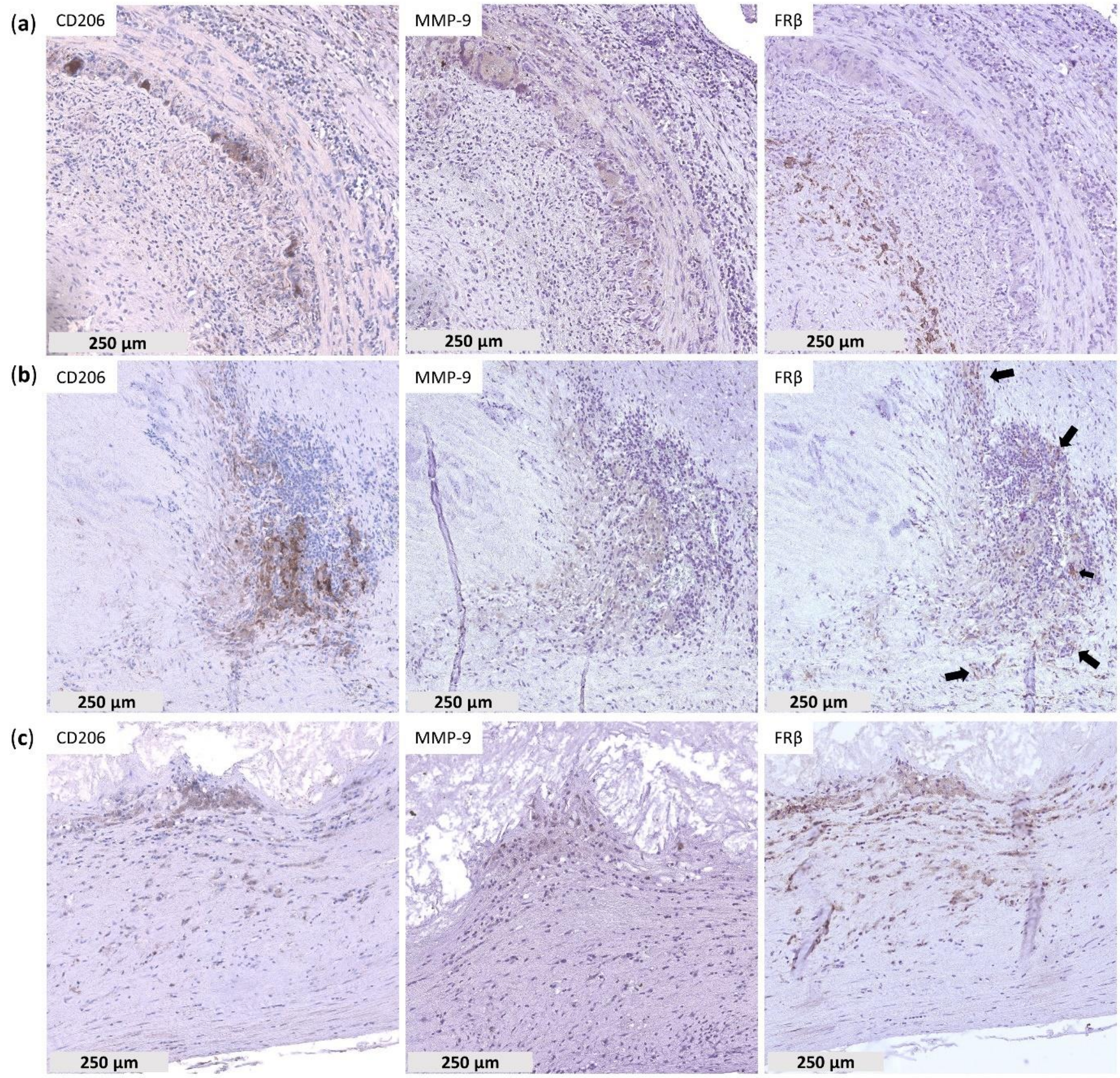

Supplementary figure 4. Distinct macrophage phenotypes are associated with tissue destruction and intimal hyperplasia in giant cell arteritis. Shown are consecutive immunohistochemical staining for CD64, IL-23, IL-12, FRβ, CD206 and MMP-9 in the GCA-affected TAB (a), GCA-affected aorta (b) and atherosclerotic aorta (c). The black arrow indicates FRβ-positive cells surround the outer region of CD206-positive rim. No distinct distribution pattern detected in atherosclerotic aortas. GCA: giant cell arteritis, TAB: temporal artery, MMP: matrix-metalloproteinase.

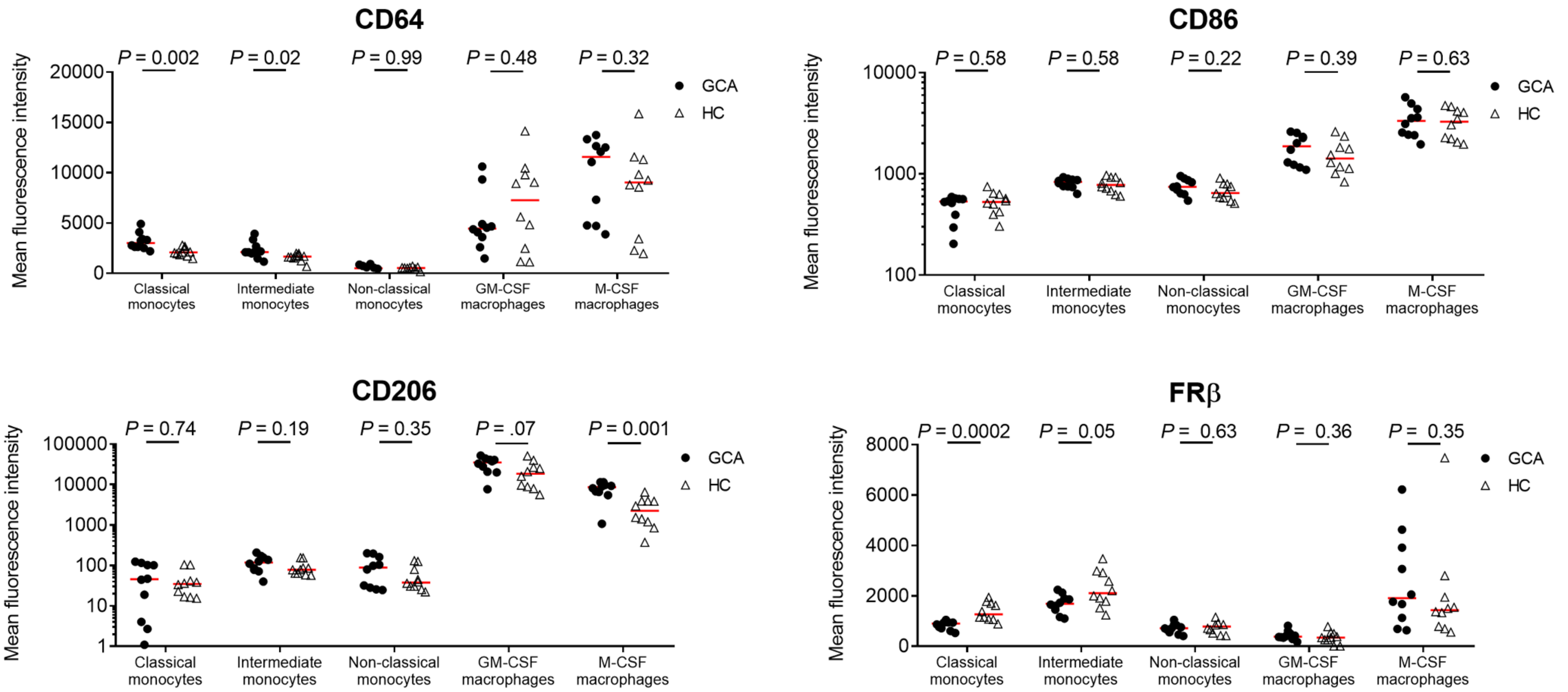

Supplementary figure 5. The effects of GM-CSF and M-CSF signals on monocyte and macrophage surface markers. Mean fluorescence intensity of CD64, CD86, CD206 and FRβ on monocyte subsets, GM-CSF-differentiated macrophages (GM-MØs) and M-CSF-differentiated macrophages (M-MØs) from GCA patients (n = 10) and healthy controls (n = 10). GCA: giant cell arteritis.

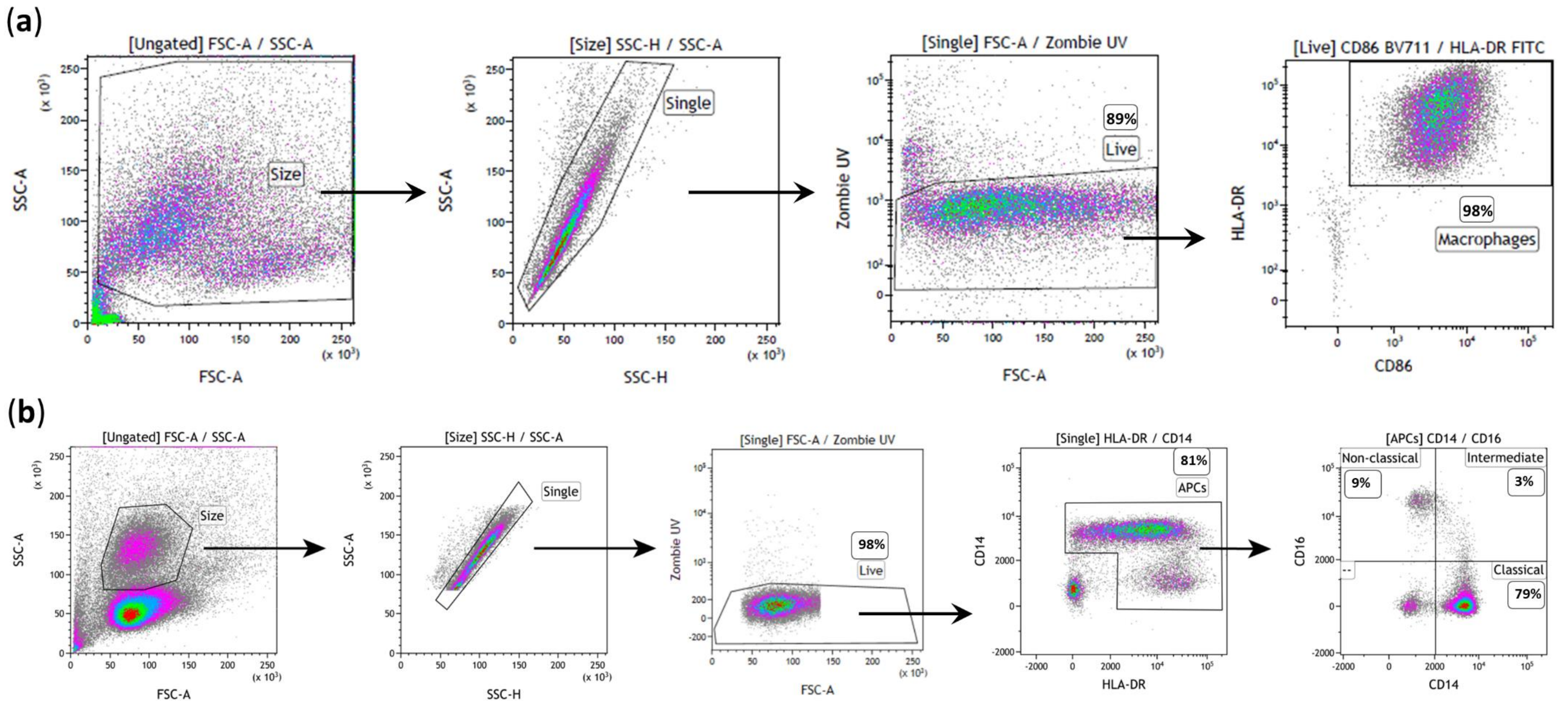

Supplementary figure 6. Gating strategies for macrophage subsets (a) and monocyte subsets (b). Shown are the representative figures of gating strategies used for gating macrophages and monocyte subsets. Monocytes and macrophages were first gated by size according to forward and side scatter (FSC/SSC). Doublets were excluded from the analysis using SSC-Height (SSC-H) versus SSC-Area (SSC-A) plots. Zombie dye-positive cells were gated out as dead cells. Contaminating lymphocytes in the monocyte gate were gated out and excluded from the analysis based on negative HLA-DR and CD14 expression. Monocyte subsets were gated based on CD14 and CD16 expression. Macrophages were gated based on positive HLA-DR and CD86 expression.
